# Nursing Interns’ Attitudes Toward, Preferences for, and Use of Diabetes Virtual Simulation Teaching Applications in China: National Web-Based Survey

**DOI:** 10.2196/29498

**Published:** 2021-09-09

**Authors:** Fang Liu, Huiting Weng, Rong Xu, Xia Li, Zhe Zhang, Kuaile Zhao, Zhiguang Zhou, Qin Wang

**Affiliations:** 1 National Clinical Research Center for Metabolic Diseases, Department of Metabolism and Endocrinology The Second Xiangya Hospital Central South University Changsha China; 2 Clinical Nursing Teaching and Research Section The Second Xiangya Hospital Central South University Changsha China; 3 Department of Ophthalmology The Second Xiangya Hospital Central South University Changsha China

**Keywords:** nursing interns, virtual simulation, China, nursing education, diabetes

## Abstract

**Background:**

Diabetes has placed heavy social and economic burdens on society and families worldwide. Insufficient knowledge and training of frontline medical staff, such as nurses, interns, and residents, may lead to an increase in acute and chronic complications among patients with diabetes. However, interns have insufficient knowledge about diabetes management. The factors that affect interns’ current level of diabetes-related knowledge are still unclear. Therefore, understanding the behavioral intentions of interns is essential to supporting the development and promotion of the use of virtual simulation teaching applications.

**Objective:**

This study aimed to identify the determinants of nursing interns’ intentions to use simulation-based education applications.

**Methods:**

From December 1, 2020, to February 28, 2021, the web-based survey tool Sojump (Changsha Xingxin Information Technology Co) was used to survey nursing interns in hospitals across China. Two survey links were sent to 37 partner schools in 23 major cities in China, and they were disseminated through participants’ WeChat networks. Multiple regression analysis was used to determine the association between demographic information and basic disease information and the use of the application for treating adult patients.

**Results:**

Overall, 883 nursing interns from 23 provinces in China responded to the survey. Among them, the virtual simulation utilization rate was 35.6% (314/883) and the awareness rate was 10.2% (90/883). In addition, among the interns, only 10.2% (90/883) correctly understood the concept of virtual simulation, and most of them (793/883, 89.8%) believed that scenario-simulation training or the use of models for teaching are all the same. Multiple regression analysis showed that the educational level, independent learning ability, and professional identity of the interns were related to use of the application (*P*<.05). Skills and knowledge that the interns most wanted to acquire included the treatment of hypoglycemia (626/883, 70.9%), functional test simulation (610/883, 69.1%), and blood glucose monitoring technology (485/883, 54.9%). A total of 60.5% (534/883) of the interns wanted to acquire clinical thinking skills, while 16.0% (141/883) wanted to acquire operational skills. Nursing trainees believed that the greatest obstacles to virtual simulation included limited time (280/883, 31.7%), the degree of simulation (129/883, 14.6%), the demand for satisfaction (108/883, 12.2%), and test scores (66/883, 7.5%).

**Conclusions:**

The understanding and usage rate of diabetes virtual simulation teaching applications by Chinese nursing interns is very low. However, they have high requirements regarding this teaching method. Conducting high-quality randomized controlled trials and designing applications that are suitable for the needs of different nurse trainees will increase students’ interest in learning and help improve diabetes knowledge among nursing interns.

## Introduction

### Background

With the rapid development of the social economy, continuous changes in modern people’s behaviors and lifestyles, and the aging of the population, the incidence of diabetes is also increasing rapidly in all parts of the world. According to forecasts, from 1995 to 2030, the number of patients with diabetes worldwide will increase from 135 million to 472 million, among which more than 75% are in developing countries [1]. According to the World Health Organization [2], as of 2015, the prevalence of diabetes in China had reached 10%, and the estimated prevalence of diabetes in adults over 18 years old in China was 11.6%. The number of adult patients with diabetes in China has reached 92.4 million [3]; China is now the country with the largest number of patients with diabetes.

Effective diabetes education for patients is indispensable; it is a necessary means to ensure that patients receive effective therapies. Nurses are the most important providers of diabetes education in China. Due to China’s national conditions, there are no specialized diabetes educators that provide health guidance and dietary education to patients with diabetes. Such work is often undertaken by clinical staff (doctors, nurses, interns, etc). Nurses and interns have the most contact with patients with diabetes and are most likely to provide patients with diabetes-related knowledge [4]. In nursing programs, Chinese students often need to go to designated hospitals for 8- to 12-month internships in their senior year. During these internships, they learn basic knowledge about diseases, professional skills, and communication skills at the hospital. At the end of the internship, they need to pass a unified examination jointly organized by the hospital and the school before they can graduate. The clinical internship is a critical period for nursing students to transition from student to nurse. At this stage, interns often cannot perform nursing activities alone, and all of these require the demonstration and guidance of clinical teachers. In most clinical teaching sessions, the teacher and the interns are apprentices. In addition to regular and unified theoretical training and operational training in the nursing department, teachers often use a one-to-one teaching mode. The importance of nursing interns in disease prevention is enormous, as they are the members of health care teams who spend the most time with the patients [5]. They also serve as resources for patients with diabetes seeking information about the early detection of diabetes complications [6]. The knowledge and practice they acquire during their studies and internships play important roles in providing accurate and up-to-date information to improve the health behaviors and outcomes of patients with diabetes. Nursing interns must possess the necessary knowledge to enable them to care for patients with diabetes, helping them to achieve a high quality of life devoid of complications [7]. In addition, an intern can detect hypoglycemia for the first time when measuring a patient’s blood sugar. If nursing interns know how to deal with hypoglycemic events, they can instruct patients to eat the correct glucose-increasing food immediately, thereby reducing the time interval from discovery to treatment of hypoglycemia and reducing the occurrence of adverse events [8].

The cultivation of self-learning ability by interns is inseparable from the application of self-learning methods and tools [9]. With the goal of improving the self-learning ability of nursing students, many scholars at home and abroad have carried out various studies in this field. Some studies have shown that project-based learning methods, preceptorship programs, and reflective diaries have improved students’ abilities for critical thinking, clinical decision making, and humanistic care [10-13]. However, these methods focus on cultivating students’ information-seeking and cooperation abilities in order to enhance the autonomous learning ability of interns, and the effect is not lasting. Without the supervision of teachers, the internal motivation of students to learn independently is still insufficient [14].

Interns’ apprenticeships have always constituted a challenge faced by the government, health educators, health managers, and the students themselves to ensure the quality and safety of learning and clinical practice [15]. Students in the 21st century are using information and communications technology (ICT) every day [16,17]. The use of ICT has led to different learning processes and information structure processes. The development of digital and virtual technology has simplified the ability to reconstruct reality using virtual patients depicted on a computer touch screen (ie, virtual simulation) [18].

A virtual simulation is a real-life reproduction depicted on a computer screen, and it involves a real person operating the simulation system. This type of simulation puts people at the center of a situation by exercising decision making, motor control, and communication skills [19]. Virtual simulation uses virtual patients in dynamic and immersive clinical environments, ranging from prehospital to community environments [20]. The latest technological advances in virtual simulation have improved their authenticity and dynamic interaction, and it is possible to display thousands of clinical situations on a touch screen or on the web [21,22]. However, little is known about their effect on students’ learning satisfaction, self-efficacy, knowledge retention, and clinical reasoning, especially when using the latest developments in virtual simulation [21].

This study aimed to assess the knowledge needs of nursing students for managing diabetes mellitus. By evaluating the self-learning ability of nursing students and the degree of demand for diabetes-related knowledge, the demand for virtual simulation teaching applications for nursing students was explored. Therefore, the purpose of this study was to evaluate the level of understanding of diabetes specialist knowledge and the demand for virtual simulation teaching among nursing students in China.

### Objectives

We aimed to investigate the use of virtual simulation teaching applications by nursing interns as well as their perspectives, attitudes, and associated factors regarding these teaching applications. We also aimed to investigate interns’ needs for these applications in order to provide information for the design of virtual simulation teaching applications and to learn how best to promote their use, which will help teachers to further improve their teaching methods and strengthen the willingness of nursing students to learn independently.

## Methods

### Questionnaire Design

An expert group consisting of five nursing educators and five clinical nursing staff members searched for applications on the national, virtual simulation, education platform; they then designed a questionnaire based on the current diabetes guidelines and the problems encountered in clinical practice. These questions were presented in a selective format. If the respondent disagreed with the listed options, they could select “other options” and write their answer in the “remarks” column. The questionnaire collected information about respondents’ demographics and their views, attitudes, and needs for virtual simulation education applications.

To determine the validity of the questionnaire content, a total of 15 experts, consisting of 12 nursing education experts and three diabetes education nurses with at least 5 years of experience, rated the relevance and clarity of the items on a 4-point scale ranging from 1 (irrelevant) to 4 (highly relevant), with a content validity index of 0.91. Before administering the questionnaire survey, we conducted a pilot test on 18 interns at Xiangya Second Hospital in China. The Cronbach α value of the questionnaire was .83.

### Survey Platform and Methods

WeChat has become one of the largest mobile traffic platforms in China. It provides many services, including messaging, free phone calls, browsing and publishing for instant sharing of information, and mobile payments [23]. It has been installed on more than 90% of mobile phones and has become part of the daily lives of most people [24]. As of 2019, the number of monthly active accounts on WeChat reached 1.15 billion, and the number of daily active accounts of mini programs exceeded 300 million [25]. As the most commonly used social media tool in China, WeChat has an expansive network of contacts. The network makes it possible for administrators to manage questionnaires through WeChat.

From December 1, 2020, to February 28, 2021, we used Sojump (Changsha Xingxin Information Technology Co), a web-based survey tool, to conduct snowball sampling through the WeChat contact network and to conduct convenience sampling through WeChat public accounts to recruit interns. The survey link was initially sent to 35 universities in 23 representative major cities in China. We asked the teachers at these universities to post the survey link on their WeChat account to reach their network contacts.

Survey respondents were all nursing trainees in China. Other nursing students who did not take part in internships at hospitals were excluded from our survey. Before administering the survey, we introduced the purpose of the survey, and the questionnaire was filled out by respondents voluntarily without any compensation.

### Ethical Approval

This study was approved by the ethics committee of the Second Xiangya Hospital, Central South University, China (ID: 2020-S790).

### Statistical Data

The data were analyzed using SPSS, version 23.0 (IBM Corp). Quantile-quantile (Q-Q) charts were used to check the normality of all continuous variables and express them as the mean (SD) or median (IQR) where appropriate. Categorical variables were expressed as frequencies and percentages. The chi-square test was used to assess the differences between groups. The generalized logic model was used to obtain the odds ratio (OR) and its 95% CI at the same time. First, we conducted a univariate analysis to analyze the OR of the potential correlation between demographic factors and autonomous learning ability. Then, we inputted all important factors into the multivariate analysis to obtain the multivariate adjusted OR. Questionnaires with missing values were excluded from the multivariate analysis. Statistical significance was defined as *P*<.05.

## Results

### Sample Characteristics

**Figure 1:**
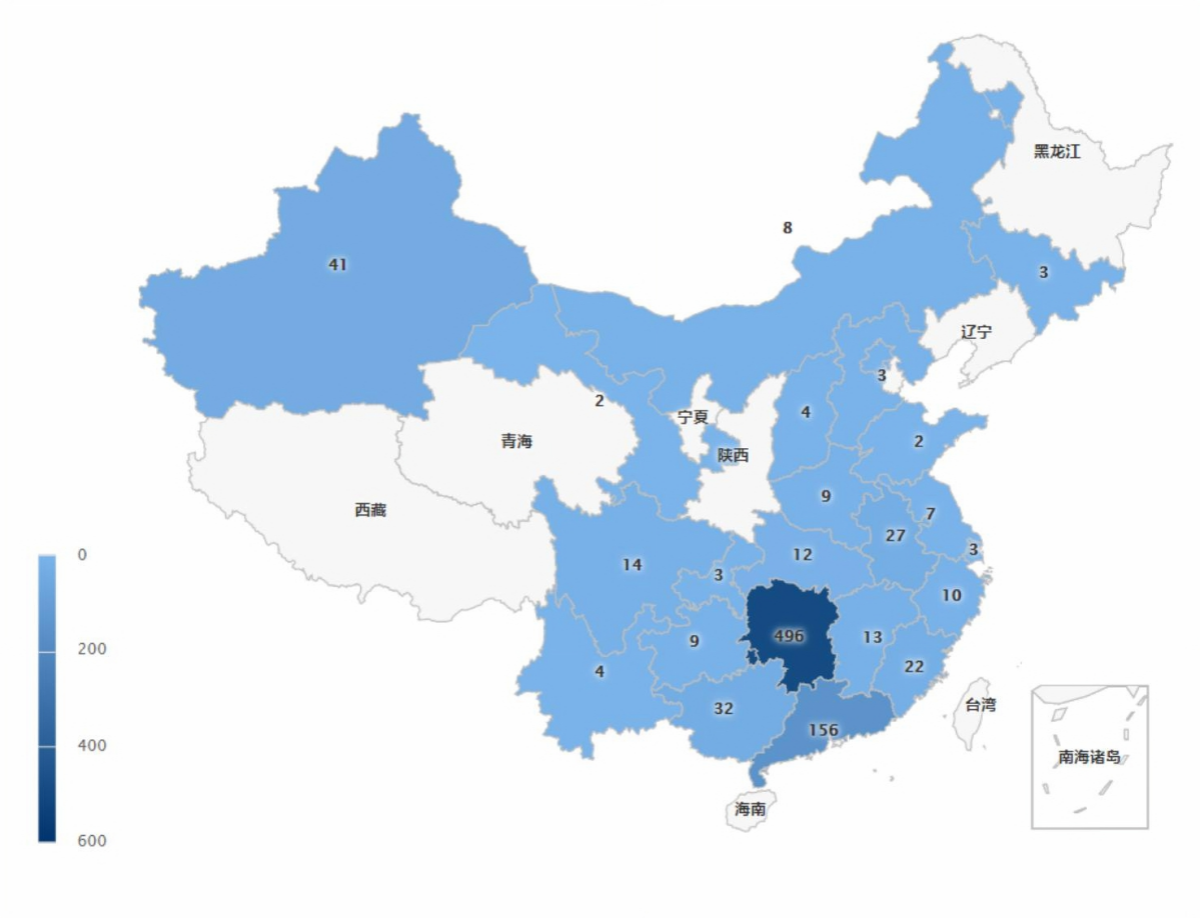
Distribution of the nursing intern sample in China by province. The numbers represent how many questionnaires were collected in each corresponding province.

A total of 883 interns distributed among 26 provinces in China (Figure 1) responded to the patient survey. The respondents’ characteristics are shown in Table 1. Among the respondents, 10.1% (89/883) were male, and respondents had a mean age of 20.64 (SD 2.1) years. Overall, 56.9% (502/883) had a bachelor’s degree. A total of 83.0% (733/883) of the respondents had been an intern for more than 8 months, and 46.5% (411/883) did not know their reason for choosing to study nursing (Table 1).

**Table 1:** Characteristics of nursing interns.

Characteristic		Respondents (N=883)
**Gender, n (%)**		
	Male	89 (10.1)
	Female	794 (89.9)
Age (years), mean (SD)		20.64 (2.1)
**Educational level, n (%)**		
	Middle school	26 (2.9)
	High school	102 (11.6)
	Technical college	502 (56.9)
	Bachelor’s degree	242 (27.4)
	Master’s degree or higher	11 (1.2)
Internship time (months), mean (SD)		6.02 (1.6)
**Reason for choosing nursing, n (%)**		
	I like nursing	287 (32.5)
	Parents’ suggestion	247 (28.0)
	Acquaintances’ recommendation	55 (6.2)
	The school transferred me	99 (11.2)
	Good employment	195 (22.1)
**Feelings about nursing, n (%)**		
	I love the nursing career	410 (46.4)
	Not sure	197 (22.3)
	I can accept as a job, but not as a career	260 (29.4)
	I don’t like nursing	16 (1.8)
**Employment intention, n (%)**		
	Nurse	747 (84.6)
	Nursing-related industries	115 (13.0)
	Others	21 (2.4)

### Assessment of the Self-Learning Ability of Interns

**Figure 2:**
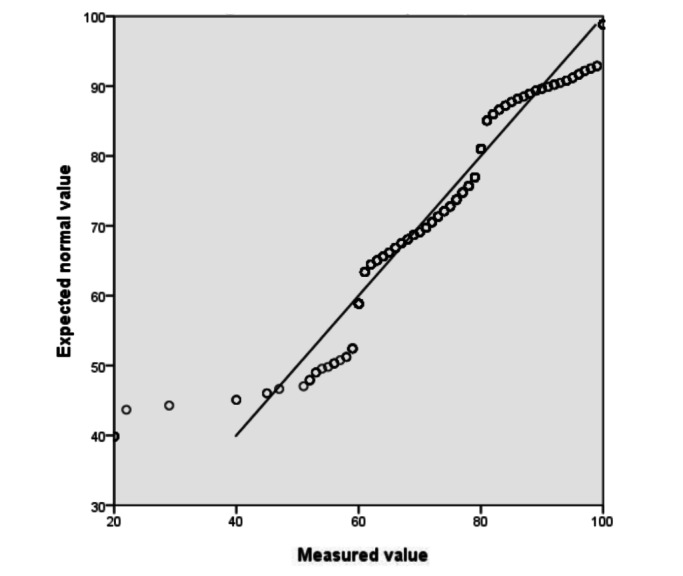
The normal quantile-quantile (Q-Q) chart for the score of autonomous learning ability.

**Figure 3:**
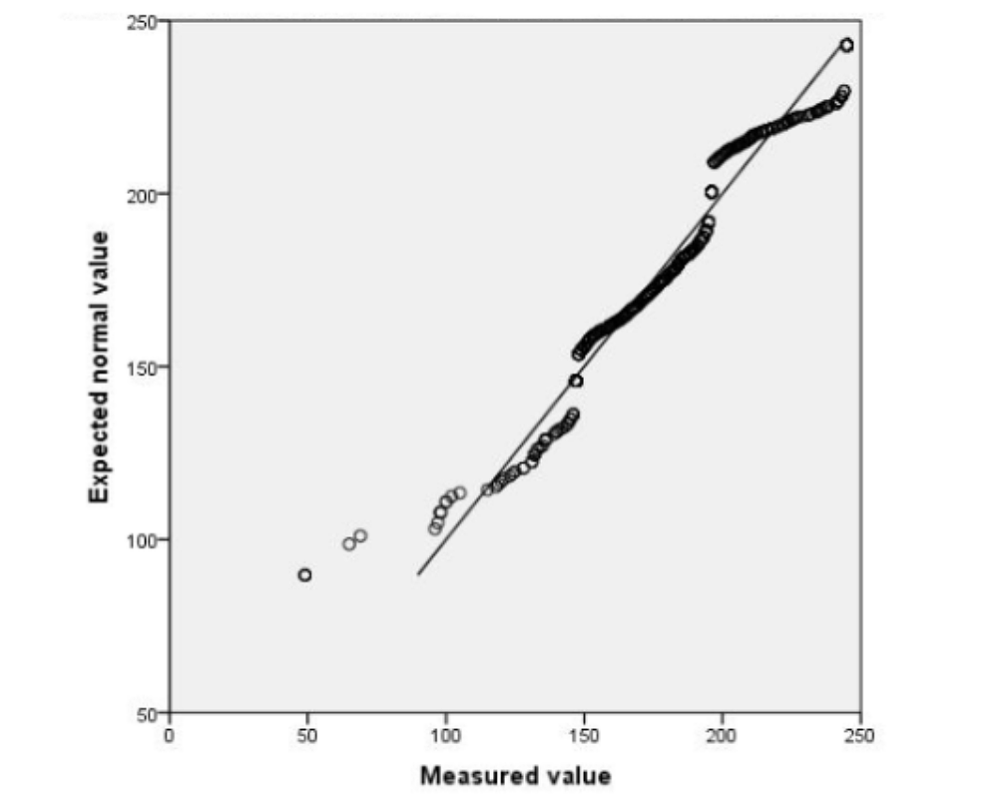
The normal quantile-quantile (Q-Q) chart for the score of the learning strategy scale.

All of the interns (N=883) were able to fill out the self-learning ability scale. The Q-Q normality was the sum of the total scores of the self-learning ability of interns (Figures 2 and 3). The total score is represented by the diagonal line, so it is considered that the total score of the autonomous learning ability of nursing students conforms to the normal distribution. The data were analyzed using the Pearson correlation coefficient. Age, gender, educational level, and length of internship were not related to the self-learning ability of interns (*P*>.05). The correlation coefficient between the “reason for choosing nursing” and the “autonomous learning ability scale score” was 0.993; the correlation between them was statistically significant (*P*<.001). This correlation was also reflected with “feelings about nursing” (*P*=.02), which showed that interns who love nursing had stronger self-learning ability. In addition, the correlation between “feelings about nursing” and the “score of the learning strategy scale” was statistically significant (*P*=.001). This indicates that the more positive feelings the nursing student interns had toward the nursing profession, the higher their scores were on the learning strategy scale (Table 2).

**Table 2:** Correlation analysis of self-learning ability of interns (N=883).

Characteristic	Autonomous learning ability		Score of learning strategy scale	
	*r*	*P* value	*r*	*P* value
Age	–0.020	.56	–0.014	.67
Gender	–0.101	.10	0.018	.59
Educational level	0.008	.81	0.008	.81
Internship time	0.410	.22	–0.044	.19
Reason for choosing nursing	0.993	<.001	0.174	<.001
Feelings about nursing	0.595	.02	0.298	<.001
Employment intention	0.011	.75	–0.175	<.001

### Interns’ Needs and Expectations of Diabetes Virtual Simulation Applications

**Figure 4:**
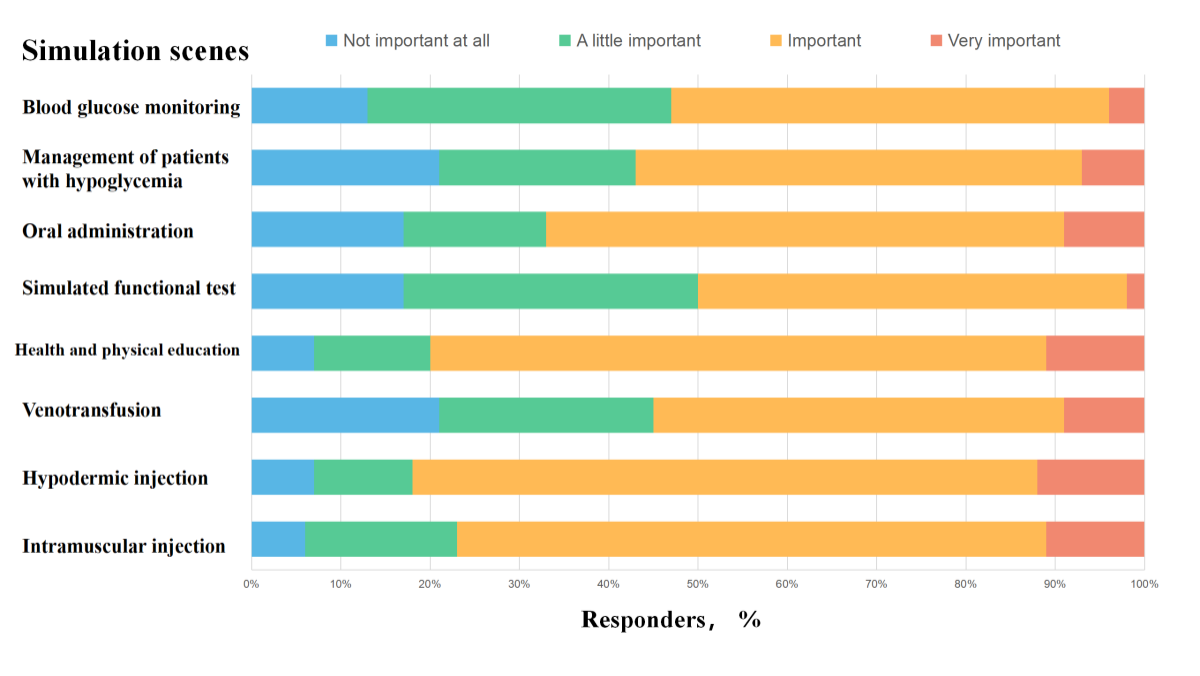
Importance of different simulation scenes on a diabetes virtual simulation application as reported by interns.

**Figure 5:**
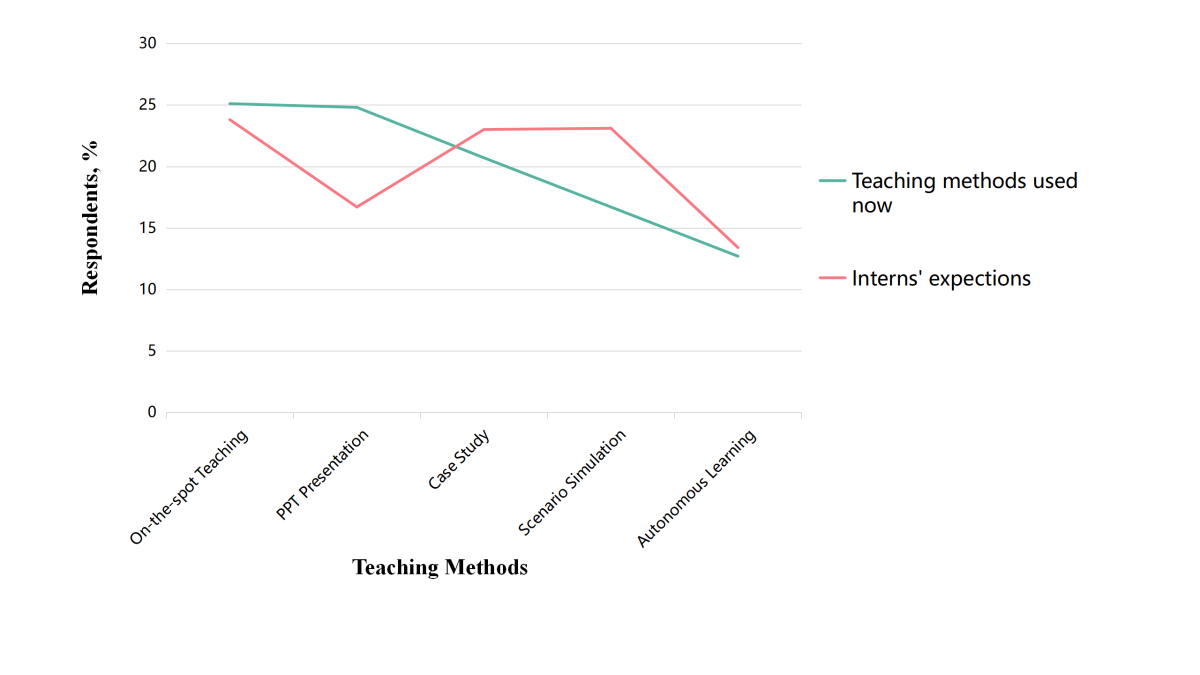
Comparison of teaching methods with interns' expectations. PPT: PowerPoint.

Nursing trainees believed that important functions of a diabetes virtual simulation application are to help them treat patients with hypoglycemia and the simulation of functional tests. Almost all respondents believed the listed functions were important or very important. However, most interns believed that oral administration, venofusion, and intramuscular injection were important (Figure 4). When comparing teaching methods with the expectations of nurse interns, PowerPoint presentations (222/883, 25.1%) and face-to-face teaching (219/883, 24.8%) were the most-used teaching methods, while students expected to use more virtual simulations (204/883, 23.1%) and to reduce the use of PowerPoint presentations (148/883, 16.8%) (Figure 5).

In this study, out of 883 interns, 569 (64.4%) had never participated in virtual simulation teaching and 793 (89.8%) had not heard of the concept of virtual simulation before this survey. Table 3 shows that through the virtual simulation application, what interns most want to improve is their clinical thinking ability (534/883, 60.5%), followed by their comprehension ability (156/883, 17.7%).

**Table 3:** Nurse interns’ usage and preferences of a diabetes virtual simulation application.

Question		Respondents (N=883), n (%)
**Have you participated in virtual simulation teaching?**		
	No	569 (64.4)
	Yes	231 (26.2)
Have you participated in similar activities? (yes)		83 (9.4)
**Do you know about virtual simulation teaching?**		
	No	793 (89.8)
	Yes	90 (10.2)
**What do you think of virtual simulation teaching?**		
	Very good	138 (15.6)
	Good	251 (28.4)
	Neutral	76 (8.6)
	Bad	7 (0.8)
	Very bad	2 (0.2)
	Do not know	409 (46.3)
**What is your acceptance level of virtual simulation teaching?**		
	Very good	354 (40.1)
	Good	393 (44.5)
	Neutral	126 (14.3)
	Bad	7 (0.8)
	Very bad	3 (0.3)
**Which ability do you most want to improve in virtual simulation teaching?**		
	Comprehension skills	156 (17.7)
	Analytical skills	141 (16.0)
	Judgment skills	48 (5.4)
	Clinical thinking ability	534 (60.5)
	Others	4 (0.5)
**What do you think is appropriate for the average duration of each session? (minutes)**		
	0-10	209 (23.7)
	11-30	483 (54.7)
	31-60	169 (19.1)
	61-90	22 (2.5)

## Discussion

### Principal Findings

#### The Use of a Virtual Simulation Teaching Application and its Influencing Factors Among Interns

Among the interns, 26.2% (231/883) had participated in virtual simulation education, and 9.4% (83/883) had participated in similar activities. These rates are comparable to results from surveys conducted in New York [17] and Florida [16], and higher than the rate (7%) found in a 2011 survey in Canada [18]. In China, more nursing interns in Southern China (87.3%) participated in virtual simulation teaching than in Northern China (12.7%). One possible reason is that China’s economic development is uneven, and medical resources are unevenly distributed. These resources are more highly concentrated in economically developed areas. In these developed areas, the economy is developing well, and the government and families attach great importance to education [26]. In addition, nursing students who participated in virtual simulation teaching preferred it (271/883, 30.7% vs 43/883, 4.9%), which is consistent with previous studies [27,28]. This could be the case because virtual simulation teaching caters to the thinking skills of young people more than traditional teaching. Through the use of virtual simulations, nursing trainees have the opportunity to practice skills and deal with difficult situations. Virtual simulation teaching allows greater access rights, and it allows interns to appear “virtually” only as participants. In addition, the virtual environment provides a safe environment for practicing nontechnical skills such as teamwork.

#### Suggestions to Promote the Use of Virtual Simulation Teaching Applications

The utilization rate of virtual simulation teaching applications in China is low because of the low awareness of this teaching method among interns. Only 10.2% (90/883) of interns had heard about virtual simulation teaching. In 2008, Tsinghua University launched a medical-related virtual simulation project for the first time to help doctors complete neurosurgery operations [29]. In 2018, China established a national virtual simulation experiment teaching platform, and virtual simulation teaching began to develop [30].

Through virtual simulation, clinical thinking was the ability that interns wanted to acquire the most (534/883, 60.5%); the second most desired ability was analytical skills (141/883, 16.0%). This result is consistent with a study in Canada [31]. This indicates that virtual simulation is a supplementary teaching strategy that provides opportunities to improve students’ clinical reasoning ability through exposure to a large number of clinical situations. The use of clinical virtual simulation as a teaching strategy should be integrated and coordinated with other teaching strategies in the classroom and other resources (eg, the high-, medium-, and low-tech simulators used in our simulation laboratory) to maximize the development of students’ cognitive, emotional, and psychomotor skills [32,33].

Nursing trainees believed that the scenarios that should be included in the virtual simulation of diabetes care are the treatment of patients with hypoglycemia (626/883, 70.9%), functional test simulation (610/883, 69.1%), and blood glucose monitoring technology (485/883, 54.9%).

Several studies have also shown that nursing interns lack the knowledge to properly handle patients with hypoglycemia, especially elderly patients with diabetes, which could increase the risk of acute complications in these patients [34,35]. This reminds us that virtual simulation is an interactive learning strategy that can increase students’ intrinsic motivation and satisfaction. It focuses on the application of basic knowledge to clinical learning challenges that reproduce the clinical scenarios that students will face in the future. It allows for competency-based education and assessment to enable deeper learning and the development of clinical expertise. Virtual simulation can help reduce clinical errors and improve the safety and quality of health care. When designing diabetes virtual simulations, we should focus on the design of scenarios for patients with hypoglycemia.

### Comparison With Previous Work

To the best of our knowledge, no large-scale survey on the use and demand of virtual simulation has been previously conducted among Chinese nursing interns. An Indian survey showed that the need for diabetes knowledge by interns is urgent, consistent with our research, but that study did not identify what kinds of teaching tools the interns wanted. The survey only investigated the needs of first-year nursing students in one city in regard to virtual simulation [36], while our research collected information about the understanding of virtual simulation among interns in various provinces of China. Our research found that students who received virtual simulation teaching tended to be younger, more educated, and have a stronger autonomous learning ability, which is consistent with a survey conducted in Canada [37].

### Strengths and Limitations

A strength of our research is that the initial survey links for patients and diabetes experts were sent to 37 partner schools in 23 representative major cities in China, and these were disseminated through their WeChat contact networks. In addition to this snowball-sampling method, the survey was also carried out through three convenience-sampling methods on WeChat Moments.

Our research also has some limitations. First, the sample of 883 nurse interns could not fully represent all interns in China. Our sample came from 23 provinces in China; thus, not all provinces were represented. Second, our sampling was not stratified by geographic area, urban or rural area, school level, or hospital level where internships were based. Certain selection biases were inevitable. Finally, our sampling was based on the WeChat network. Although WeChat has 1.04 billion monthly active users [38], some people rarely use WeChat or surf the internet. Our research methods included a cross-sectional survey. Although the views and attitudes of interns are very important in developing teaching methods for them, people’s attitudes toward the usefulness of simulations and their possible effects depend to a large extent on their current technological development and implementation methods. Therefore, with the development of technology and changes in people’s perceptions, these findings must be updated over time. In addition, many factors affect the use of teaching methods. Although we adjusted for some factors in the multivariate analysis, other potential confounding factors still exist.

### Conclusions

Chinese nursing interns’ awareness and usage of diabetes virtual simulation teaching methods are low. However, interns desire the knowledge they would gain by using these methods. Designing virtual simulations of diabetes that are suitable for the needs of different nurse trainees will increase students’ interest in learning and help improve diabetes knowledge among nursing interns. High-quality randomized controlled trials can be conducted to improve the effectiveness of virtual simulation teaching of diabetes, provide evidence for teachers to choose suitable teaching tools, and help with the promotion of the correct management of diabetes. China should improve people’s understanding of virtual simulation teaching in universities, and relevant policies and regulations should be published to support teachers in using virtual simulation teaching tools in schools or hospitals. Virtual simulation is a potentially effective supplement for teaching. It can be used anywhere and at any time to improve the self-learning methods of Chinese nursing interns.

## Abbreviations

ICT: information and communications technology
OR: odds ratio
Q-Q: quantile-quantile
